# Impulsivity as a Risk Factor for Suicide in Bipolar Disorder

**DOI:** 10.3389/fpsyt.2021.706933

**Published:** 2021-07-23

**Authors:** Przemysław Zakowicz, Maria Skibińska, Karolina Wasicka-Przewoźna, Bartosz Skulimowski, Filip Waśniewski, Aneta Chorzepa, Maciej Różański, Joanna Twarowska-Hauser, Joanna Pawlak

**Affiliations:** ^1^Department of Psychiatric Genetics, Poznan University of Medical Sciences, Poznan, Poland; ^2^Center for Child and Adolescent Treatment in Zabó, Zielona Góra, Poland; ^3^Department of Child and Adolescent Psychiatry, Poznan University of Medical Sciences, Poznan, Poland; ^4^Department of Adult Psychiatry, Poznan University of Medical Sciences, Poznan, Poland

**Keywords:** bipolar disorder, suicide, impulsivity, neuropsychology, personality, risk factors

## Abstract

The accurate assessment of suicide risk in psychiatric, especially affective disorder diagnosed patients, remains a crucial clinical need. In this study, we applied temperament and character inventory (TCI), Barratt impulsiveness scale 11 (BIS-11), PEBL simple reaction time (SRT) test, continuous performance task (CPT), and Iowa gambling task (IGT) to seek for variables linked with attempted suicide in bipolar affective disorder group (*n* = 60; attempters *n* = 17). The main findings were: strong correlations between self-report tool scores and objective parameters in CPT; the difference between attempters and non-attempters was found in the number of correctly responded trials in IGT; only one parameter differed between attempters and non-attempters in BPI diagnosis; and no significant differences between suicide attempters and non-attempters in TCI, BIS-11, and SRT were found. These justify the conclusion that impulsivity itself is not a strong predictor, and used as a single variable might not be sufficient to indicate the high suicide risk group among bipolar patients.

## Introduction

Every 40 s one person dies by suicide that is ~800,000 people every year worldwide (1).

The most important risk factors are: barriers in access to health care; access to lethal means, trauma, or abuse; sense of isolation and lack of social support; previous suicide attempts; mental disorder; and family history of suicide (2). Those suffering from psychiatric disorders have significantly higher risks of suicide, especially in the first few months after diagnosis (3). Over 90% of individuals, who have committed suicide, fit the criteria for having a mental health problem (4). In bipolar affective disorder, 20% of patients commit suicide (5), and 25–50% of them present suicidal attempts during the illness (6). For bipolar disorder, the following suicide risk factors were identified: rapid-cycling course, mixed episodes or agitated depression, early-onset of the disease, and comorbidity with anxiety and substance use disorders (4), and also the period soon after hospital discharge (7). However, previous assessment tools used to recognize suicide risks have shown to be challenging (3) and are characterized by unsatisfactory sensitivity and specificity (8).

Apart from the current medical burden and social stressors, personality traits (9) have been investigated as distal risk factors (10, 11). The influence of decision-making includes personality traits, such as aggression, anger, hostility, emotional instability, and impulsivity (12).

Impulsivity plays an important role in predisposing to psychiatric illnesses, such as bipolar disorder, depressive disorder, behavioral and substance addictions, and personality disorders (13–15). According to diagnostic and statistical manual of mental disorders (DSM-5), impulsivity is “acting on the spur of the moment in response to immediate stimuli; acting on a momentary basis without a plan or consideration of outcomes; difficulty establishing and following plans; a sense of urgency and self-harming behavior under emotional distress. Impulsivity is a facet of the broad personality trait domain—disinhibition.” Whiteside and Lynam (16) have described four subdimensions of impulsivity: urgency, lack of premeditation, lack of perseverance, and sensation seeking, whereas, Barrat impulsiveness scale subdivides impulsivity into three factors (17) subdivides impulsivity into three factors.

Brain structures involved in impulsive behavior are the orbitofrontal cortex (18), the anterior cingulate cortex, the infralimbic cortex (19), and the dorsolateral prefrontal cortex (20). Premature responses strictly corresponded with dopamine and serotonin level (21).

Patients with bipolar disorder I especially present high levels of impulsivity (15, 22). Interestingly, high impulsivity is present not only during manic or depressive episodes but also in patients with euthymia (23). Rote et al. (24) found that attentional impulsivity was higher in patients with euthymia than healthy controls and seems to be a predictive factor for severity of the illness (24). Impulsive action, especially combined with anger-related traits and novelty seeking, is a risk factor for suicidal behaviors (25). Perraud et al. (26) found that suicide attempters characterize with higher harm avoidance and novelty seeking and lower self-directedness than non-attempters. High novelty seeking presents as excessive anger, quick decision-making, and poor impulse control. These features may predispose to a particular type of suicidality (26), namely to multiple attempts and first attempt at a younger age. Relatively lower impulsivity in the attempter group was connected with higher lethality of suicide behavior (27). Other researchers found that high impulsiveness predisposes to choosing violent suicide methods (28). Although impulsivity has been associated with suicidal behavior (29) and plays a vital role in understanding many psychiatric disorders, such as mania, substance abuse, personality disorders, or attention deficit hyperactivity disorder (ADHD) (30), the results of clinical investigations are inconsistent in the field of suicidality. Impulsivity can be measured with methods based on self-reporting, such as BIS-11, Temperament and Character Inventory (TCI), or with behavioral tasks. Recently, behavioral tasks have become preferred to self-report tools because they allow to objectively measure different aspects of impulsivity. Studies demonstrate that self-report and behavioral measures of impulsivity can show independent results, not correlating with each other (31).

The aim of this study is to search for a potential auxiliary method to assess the individual predisposition of patients to act upon suicidal ideas. We hypothesize that suicide attempters differ from non-attempters in impulsivity parameters and that the trait is detectable as an intermediate phenotype. We used measurement of several aspects of impulsivity to seek its link to suicide behavior in bipolar affective disorder. Results of subjective (self-report questionnaires) and objective (computerized performance tasks) methods were compared. Subsequently, we analyzed the obtained scores in suicidal and non-suicidal patients.

## Materials and Methods

### Subjects

The investigated group included only patients diagnosed with BP (*n* = 60; 21 men, 39 women). As inclusion criteria, we used the diagnosis of bipolar affective disorder, age 18–70 years and ability to perform computerized tasks, being right-handed, and lack of severe somatic and neurological problems that require immediate medical intervention. Co-occurrence of BP with other axis I disorders or personality disorders was not an exclusion criterion. The diagnosis was established with SCID-I questionnaire (The Structured Clinical Interview for DSM-IV Axis I Disorders) (32) according to DSM-IV criteria. All patients had a history of at least one in-patient clinic treatment when the diagnosis was confirmed. Data on suicidality, illness duration, and family burden were completed in an additional interview. Beck Depression Inventory, Young Mania Rating scale, and Hamilton Depression Rating scale were used to confirm the euthymic state in time point of neuropsychological computerized assessment, BIS-11, and TCI completion.

Suicide attempt was defined as self-destructive behavior of an individual with some intention to end life by himself/herself (33). About 17 patients have had a history of suicidal attempts, among them eight used violent methods [hanging, firearms, jumping from a height, deep cuts, car crash, burning, gas poisoning, drowning, electrocution, and jumping under a train according to Asberg et al. (34); Ludwig and Dwivedi (35)], and 10 individuals did not provide information about the history of suicidality, presence of thoughts, and the number or method of an attempt.

The patients received a detailed description of the study procedures and gave informed written consent for participation in the study. The protocol was approved by the Ethics Committee, Poznan University of Medical Sciences.

### The Personality and Neuropsychological Assessment

One of the most commonly used self-report measures is the BIS-11 (17), which subdivides impulsivity into factors and subfactors: (1) attentional: attention; cognitive instability; attentional total; (2) motor: motor perseverance; motor total; and (3) non-planning—self-control; cognitive complexity; non-planning total. The TCI (36) is an instrument providing a deep and comprehensive model of personality. It deconstructs personality into seven dimensions (36, 37). Here, impulsivity is a subdimension of novelty seeking.

The other methods of assessing impulsivity are objective behavioral tasks, which are often computerized. They are presented in the form of games, in which the strategy, general score, and reaction time of the patient are measured. There are numerous tests that are relevant to assess reactivity, ability to inhibit action, and decision-making styles, such as simple reaction time (SRT) test, continuous performance task (CPT), and Iowa Gambling Task (IGT).

The personality and neuropsychological assessment was performed in euthymic state (<8 points in the Beck Depression Inventory; <6 points both in Young Mania Rating scale and Hamilton Depression Rating Scale). Personality traits were depicted using TCI; total dimension scores and subdimensions were used for analyses. Impulsivity was also measured using BIS-11 and again, factors and subfactors of the scale were analyzed.

To objectively measure the impulsive behavior of patients, we used the computerized version of the SRT test, CPT, and IGT from the psychology experiment building language (PEBL) battery (38). All computer tests were performed in the same order between 9:00 a.m. and 12:00 p.m.

In the SRT, the patient was presented with a visual stimulus and was asked to respond as quickly as possible (39). In CPT, the patient was to respond to many letters, but the stimulus for which the patient should inhibit the action and not respond was the letter “X.” The IGT is a test in which the patient is asked to maximize his/her profit through selection of cards from four decks. Cards from different decks are associated with different fines and rewards. Through the trial-and-error method, the patient should strive for optimal strategy (40).

### Statistical Analysis

The distribution of the data was analyzed using the Lilliefors test. Nonparametric tests were applied. The Mann–Whitney *U*-test was used in the comparisons of the results of self-report questionnaires and computerized performance tasks with dichotomous variables (suicide attempts, diagnosis, and gender).

Spearman's rank correlation coefficient was performed. Power analysis was done using the G^*^Power program (https://www.psychologie.hhu.de/arbeitsgruppen/allgemeine-psychologie-und-arbeitspsychologie/gpower) (41). We present the raw data with power analysis and without multiple-testing correction to avoid omitting clinically important, but statistically insignificant results. The significance level was set at *p* < 0.05. Analyses were made using the STATISTICA 13.3 (StatSoft, Krakow, Poland).

## Results

### Descriptive Results

The demographic structure and family burden of psychiatric disorders of the investigated group is presented in Table 1. The duration of illness was 1–49 years (mean 16.593 years; SD 11.703). The study group consisted of 39 patients with bipolar type I and 21 with type II. Suicide attempters and non-attempters groups varied in gender structure.

**Table 1:** Demographic structure of studied population.

	**Bipolar (BP) affective disease**			**BPI**	**BPII**	***p***
	**General**	**Male**	**Female**			
Number	60	21	39	39	21	0.026
Mean age (±SD)	46 (±14.24)	47 (±13.96)	45 (±14.52)	47 (±12.32)	43 (±17.36)	0.687
Mean age of disease onset (±SD)	29 (±11.73)	27 (±12.76)	30 (±11.19)	27 (±9.56)	32 (±14.60)	
Family History of psychiatric disease	45	18	27	29	16	
Family history of affective disease	18	4	14	9	9	
	**Suicide attempters**		**Suicide non-attempters**			
Number	17 (men:2; women:15)		43 (man:16; women:17)			0.013
Mean age (±SD)	44 (±16.27)		46 (±13.52)			0.315
Family history of suicide attempts	3		6			
Family history of committed suicide	3		7			
Diagnosis	BPI: 6 BPII: 11		BPI: 33 BPII:10			

### Suicide Attempters vs. Non-suicide Attempters

In the entire bipolar group, the Mann–Whitney *U*-test did not detect any significant differences between suicide attempters and non-attempters in TCI, BIS-11, SRT test, and IGT. Suicide attempters achieved higher scores in attentional factor of BIS-11 scale than non-attempters; however, only a statistical trend was detected (*p* = 0.063).

In CPT parameters, the difference between attempters and non-attempters was found in the number of correctly responded trials (corr trials). Non-attempters obtained a higher score than the attempters (*p* = 0.040).

Then, the analysis for the BPI subgroup was performed. The computations for the BPII subgroup were omitted due to a low number of participants. Analysis of the BPI suicide attempters vs. suicide non-attempters revealed significant differences in TCI empathy subdimension (C2 score in cooperativeness), (*p* = 0.003). Suicide attempters achieved higher scores in this variable. The results of the BIS-11 scale did not differ between BPI attempters andnon-attempters.

The CPT correct response mean time was found higher for suicide non-attempters (*p* = 0.019). Another studied parameter of CPT did not occur to be significantly different. No differences were found in SRT results. Regarding the results of IGT one parameter differed between attempters and non-attempters in the BPI diagnosis. It was the number of A deck choices in the fifth block. Non-attempter have chosen this deck (less advantageous to achieve the goal of the task) more often in the last block than attempters (*p* = 0.012; see Table 2).

**Table 2:** Significant differences between suicide attempters and non-suicide attempters in the Bipolar I + II group and in Bipolar I subgroup.

	***U***	***Z***	***p***	**Power**
**BPI** **+** **BPII**				
BIS-11 attentional	116	1.857331	0.t063	0.61
CPT corr trials	162	−2.04798	0.040	0.39
**BPI**				
TCI cooperativeness C2	28.5	2.81174	0.005	0.99
CPT corr RT mean	53	−2.30717	0.021	0.4
IGT nA5	56	−2.47173	0.013	0.9

*Mann–Whitney U-test*.

### Comparisons of The BPI and BPII Groups

Between patients diagnosed with BPI and BPII, we found no differences in the BIS-11 and TCI scales (*p*-value ranged 0.074–0.992). Regarding the neuropsychological tests, significant differences between BPI and BPII were obtained for PEBL-CPT in the following parameters: the number of correctly responded trials (corr trials) (*p* = 0.003), the rate of correctly responded trials in the whole number of trials to respond (targ acc rate) (*p* = 0.004), rate of inhibited reactions in the whole number of trials to inhibit (foil acc rate) (*p* = 0.011), early response number (commission errors) (*p* = 0.008), lack of required response number (omission errors) (*p* = 0.004), and mean response time in correct responses (Corr RT mean) (*p* = 0.019). For the SRT test, the significant difference was observed in the number of early reactions (anticipations), and the patients with BPI more often responded before the stimulus (*p* = 0.008; Table 3).

**Table 3:** Comparison of CPT and SRT results between Bipolar I and Bipolar II patients.

	***U***	***Z***	***p***	**Power**
**CPT**				
Corr trials	185.5	−2.9624	**0.003**	0.36
Targ acc rate	189	−2.90177	**0.004**	0.35
Foil acc rate	209	−2.55529	**0.011**	0.61
Commission errors	203.5	2.65057	**0.008**	0.71
Omission errors	191.5	2.85846	**0.004**	0.34
Corr RT mean	221	2.3474	**0.019**	0.54
Error RT mean	283	1.27331	0.203	0.37
Sensitivity	308	0.84021	0.401	0.08
**SRT**				
Anticipations	234	2.604231	**0.009**	0.52
Delayed responses	393	−0.08707	0.930	0.05
Mean RT	302	1.52771	0.127	0.39
Median RT	310.5	1.393145	0.164	0.31

*Mann–Whitney U-test. Bold values represent statistical significance*.

Male and female groups were compared. We obtained no significant difference in BIS-11 factors and subfactors, CPT, and SRT parameters. TCI scores were significantly different between men and women regarding the following personality dimensions: anticipatory worry, a subdimension of harm avoidance (Ha1) was higher in women (*p* = 0.040); sentimentality, a sub-score of reward dependence (Rd1) was higher in women (*p* < 0.001); and empathy, a subdimension of cooperativeness (C2) was higher in women as well (*p* = 0.023).

The only objectively measured variables that differ between men and women were observed in IGT. The test was split into five blocks with 20 card choices in each block. We observed the changes of parameters of decision-making between blocks. Significant differences were noticed in the sum of advantageous and disadvantageous decks chosen in blocks 3, 4, and 5. Women chose advantageous decks more often (*p* = 0.003, 0.006, and 0.002, respectively). The difference was observed in the number of deck B and D choices in block 5. Men more often chose deck B, and women more often chose deck D (*p* = 0.019 and 0.019, respectively). The trend for more advantageous choices was found in the total sum of advantageous and disadvantageous decks: women had chosen C and D decks more often (*p* < 0.001) (Table 4).

**Table 4:** Significant differences in the Temperament and Character Inventory (TCI) and the Iowa Gambling Test (IGT) between women and men.

	***U***	***Z***	***p***	**Power**
TCI harm avoidance HA1	198	2.05537	0.040	0.736
TCI reward dependence RD1	135.5	3.273	0.001	0.999
TCI cooperativeness C2	188.5	2.24045	0.025	0.91
blok3_sum IOWA	215	2.90502	0.004	0.59
blok4_sum IOWA	226.5	2.72296	0.006	0.51
blok5_sum IOWA	204	3.07917	0.002	0.75
nB5 IOWA	251	−2.3351	0.020	0.97
nD5 IOWA	251	2.3351	0.020	1.0
Total sum of IOWA	185	3.37996	0.001	0.79

*Mann–Whitney U-test*.

### Correlations of Parameters Obtained in Subjective and Objective Impulsivity Measure Methods

We used Spearman's rank-order correlations method to search if parameters of TCI, BIS-11, and neuropsychological tests are correlated and if the correlation is positive or negative. The comparison showed a large number of correlations but was in majority *R* < 0.6. The most significant correlations with *R* > 0.4 are depicted in Supplementary Table 1. With a given sample size, power >0.9 was achieved with *R* > 0.45. The strongest correlations were revealed between Ns3 subdimension and motor BIS-11 factor (*R* = 0.609), between total Ns dimension score and motor BIS-11 factor (*R* = 0.640), and between Ha1 and BIS-11 cognitive instability score (*R* = 0.606). The correlations between self-report tool scores and objective parameters were weak. The highest observed correlations were presented between Ns4 disorderliness and median RT (from SRT) with *R* = -0.553.

## Discussion

In this study, we present an analysis of subjective and objective methods in impulsivity assessment, comparing its results with the individual history of suicide attempts among patients with bipolar disorder. This study design was inspired by the clinical need for a short, easy to apply and objective tool to assess suicide risk. We took into account that suicide has a strong biological root (10, 42, 43) and personality traits may serve as intermediate phenotypes (44).

The main aim of this study was to compare impulsivity parameters of suicide attempters and non-attempters in the bipolar population and seek for variables potentially indicating high-risk patients. We found that patients with BPI with suicidal history tend to present with higher empathy (C2) and in a trend toward higher attentional impulsivity in subjective assessment. In objective assessment with the use of the PEBL neuropsychological tasks, we found a higher rate of correct trials in CPT and correct response mean time for suicide non-attempters and a higher number of risky choices in the fifth block of IGT (nA5).

Available data strongly emphasizes that impulsivity impacts the course of bipolar disorder (45–47); however, regarding the use of BIS-11, results are inconsistent. Swann et al. (47) found the total score of BIS-11 to be significantly higher in bipolar suicide attempters, but more recent data did not confirm these results (48, 49). Our findings suggest that several components of impulsivity (namely attentional factor of BIS-11) may be involved in higher suicidal risk. Other researchers observed that bipolar patients present with an impulsive behavior rather due to impaired emotional regulation (emotion-triggered impulsivity) (50) than as the effect of altered executive functions. Watkins and Meyer (49) pointed that further analysis of the relationship between impulse control and suicidality should include other variables potentially influencing suicidality, such as personality traits, or substance use. There are two main approaches in the research of the role of impulsivity in suicide risk. A prospective observation of a cohort of patients [2-year follow-up study by Oquendo et al. (46)] confirmed the link between higher impulsivity and the incidence of suicidal acts. Another approach is broadening the tools in impulsivity assessment (behavioral tasks) and comparing it with personality traits as presented in this study.

Previous studies indicated that the IGT score significantly correlates with the history of suicide attempts (3). Using IGT, many studies have shown that suicide attempters present with a tendency to riskier choices, especially when the attempt was carried out using a violent mean [see a meta-analysis (51)].

In the current investigated group, we obtained results of IGT that were not fully in line with those mentioned above. Suicide non-attempters with BPI diagnosis significantly more often choose disadvantageous cards (A deck) in the late phase of the test than attempters. This could be interpreted as a stronger tendency in attempters to avoid frequent losses regardless of the total gain. Suicide attempters may also be less responsive to short-time gains offered in the deck A. Our results may be biased due to the low sample size. Cognitive load and a high number of information processing simultaneously (dividing attention) may impact the awareness of gains and losses and also the preference of deck (52).

In CPT, a higher commission error score was found in patients with bipolar disorder compared to healthy controls, with medium to large effect sizes (53). CPT commission errors were associated with the risk of suicide attempt in mood disorders. The authors “suggested that CPT performance is more closely associated with mood disorders than suicidal behavior” (54). The study by Keilp et al. (55) revealed no significant difference between suicide attempters and non-attempters in discrimination index *d*′ [based on total commission and omission errors (56)]. In this study, we obtained significant differences in CPT (Corr RT mean) depending on the suicide history, contrasting with the results of Keilp et al. (55).

Personality traits were identified as a marker of suicidal risk (9, 57). Higher harm avoidance and mood disorder diagnosis were strong predictors of suicide attempt (58). In the term of personality traits, we obtained higher cooperativeness among suicide attempters, what needs to be thoroughly analyzed. Moreover, we observed higher scores of cooperativeness in suicide attempters than in non-attempters. Conversely, Jylhä et al. (59) indicated that low cooperativeness, low self-directedness, low reward-dependence, and high self-transcendence were associated with suicide. Other studies indicated also a higher harm avoidance and a lower persistence, with significantly lower cooperativeness in character inventory part (60). The study by Pawlak et al. (61) confirmed novelty seeking and harm avoidance to be associated with the suicide risk among bipolar patients, whereas cooperativeness appeared to play a protective role.

Currently, we obtained numerous correlations among BIS-11 and TCI scores of the self-description scales, but only a few of them were strong *R* > 0.6. A high correlation rate (*R* > 0.6) was discovered for motor BIS-11 and extravagance (Ns3) and for total novelty seeking and motor BIS-11. Similar correlations were found in literature in which research groups diagnosed substance use disorders, pathological gambling, and sexual addiction (62–64). The BIS-11 total score was significantly correlated with all TCI domains, excepting persistence, among patients with cocaine addiction (62). The authors have rarely immediately compared BIS-11 and TCI scores. The BIS-11 non-planning impulsivity score negatively correlated with the impaired IGT in alcohol-dependent subjects, but no strong correlations with TCI were reported (63). Co-occurrence of both several personality traits and impulsivity may promote risky behaviors in patients with bipolar disorder. The data indicate that several tools measure non-fully overlapping parameters.

In the study, we attempted to distinguish between patients with BPI and BPII using TCI scores.

We found no differences between patients with BPI and BPII in subjective impulsivity assessment using BIS-11 and TCI. Recent study by Izci et al. (65) showed significant differences between BPI and BPII in BIS-11, namely in attention scores (higher in BPII) and motor and non-planning impulsivity scores (higher in BPI).

An explicit distinction was noticed in the objective, behavioral measurement: CPT and SRT. Patients with BPI occurred to present more deficits in maintaining sustained attention and higher attentional impulsivity than BPII patients. Analysis of SRT outcomes evidenced that patients with BPI differed with a higher rate of precocious reactions, whereas both CPT and SRT performance was more disturbed in the BPI group.

There is a lack of large-scale data assessing executive function differences between patients with BPI and BPII. The current approach underlines clinical (66) and genetic (67) distinction between both diagnoses, BPI and BPII that may also vary in decision-making and the impulsivity rate (68). These distinctions may impact suicidality. Our results are partially consistent with the study of Kung et al. (68) and obtained with the use of Conner's CPT-II. Here, we present the analysis performed with a similar number of patients, but a different set of variables revealed to be affected. The differences were elicited in omission errors, foil accuracy rate, and inhibition of the response on presented stimulus (correctly responded trials and target accuracy rate).

## Conclusion

We conclude that differences between BPI and BPII in TCI, and—in terms of impulsivity—in BIS-11, are not strong enough to distinguish between diagnoses. Objective measurements showed that clinically more severe type I disorder presents with worse performance in neuropsychological tasks. According to Akiskal's theory of bipolar spectrum (69), subtypes vary in terms of clinical traits and biological background and also in suicidality with a higher prevalence for BPII disorder (70).

Differences in neuropsychological features may be biased owing to the result of sex differences. Women in our studied population tended to have a safer decision-making style and higher anticipatory worry (Ha1), sentimentality (Rd1), and empathy (C2) (data not shown). Subdivision of attempter group regarding the sex was applied, and the comparison between male and female attempters and non-attempters revealed significant differences. Suicide attempters vary from non-attempters in nA5 IGT results obtained in the entire attempters group. However, the range of results in the female attempter subgroup clearly overlaps with those obtained by female non-attempters. This example illustrates that translating statistically significant difference into clinical meaning remains challenging. We decided not to discuss all these results because of the highly limited subgroup size (female attempters *n* = 15, male attempters *n* = 2), which limits its reliability. The results should be interpreted with caution. In consequence, we did not find that suicide attempters present with higher impulsivity than non-attempters in BIS-11, possibly due to male underrepresentation in the attempters group. Available data suggest higher gambling behaviors among men (71). Undertaking risky activities, like substance abuse, may predispose to higher self-directed violence and concomitantly increase the risk of suicide in men (72).

This research confirmed that impulsivity is not the only factor increasing the risk of suicide in bipolar patients; however, it may play a significant role as the cofactor among numerous traits that constitute the risk. Moreover, subjective methods, based on self-reporting used in impulsivity assessment, like BIS-11, may not provide the clear distinction between attempters and non-attempters.

Searching for adequate tools to indicate the patients at risk for suicide remains an important field of study. The role of neuropsychological traits requires further investigation. CPT and IGT provide promise, but further investigations are needed. Moreover, a prospective study would give better insight into cause-and-effect relationships. An additional important step would be the research on biological correlates of neuropsychological variables. Our study is not free from limitations including (i) the number of study participants, (ii) lack of analysis between age and personality traits, (iii) potential influence of long-lasting experience of psychiatric disease and life-threatening situations caused by suicidal attempts on personality features, and (iv) the study shows cross-sectional, not longitudinal observation.

## Data Availability Statement

The original contributions presented in the study are included in the article/Supplementary Material, further inquiries can be directed to the corresponding author/s.

## Ethics Statement

The studies involving human participants were reviewed and approved by Poznan University of Medical Sciences, Ethics Committee. The patients/participants provided their written informed consent to participate in this study.

## Author Contributions

JP, PZ, and JT-H: conceptualization. MS and JP: methodology. PZ, KW-P, BS, and FW: manuscript preparation. MR and JP: investigation. All authors contributed to the article and approved the submitted version.

## Conflict of Interest

The authors declare that the research was conducted in the absence of any commercial or financial relationships that could be construed as a potential conflict of interest.

## Publisher's Note

All claims expressed in this article are solely those of the authors and do not necessarily represent those of their affiliated organizations, or those of the publisher, the editors and the reviewers. Any product that may be evaluated in this article, or claim that may be made by its manufacturer, is not guaranteed or endorsed by the publisher.

## References

[1] Ritchie H Roser M Ortiz-Ospina E. Suicide. Published online at OurWorldInData.org (2015). Available online at: https://ourworldindata.org/suicide

[2] Saxena S Krug EG Chestnov O World Health Organization. (editors). Preventing Suicide: A Global Imperative. Geneva: World Health Organization (2014).

[3] Bolton JM Gunnell D Turecki G. Suicide risk assessment and intervention in people with mental illness. BMJ. (2015) 351:h4978. 10.1136/bmj.h4978 26552947

[4] Wasserman D Rihmer Z Rujescu D Sarchiapone M Sokolowski M Titelman D. [The European Psychiatric Association (EPA) guidance on suicide treatment and prevention]. Neuropsychopharmacol Hung. (2012) 14:113–36. 10.1016/j.eurpsy.2011.06.003 22710852

[5] Malafosse A. Genetics of suicidal behavior. Am J Med Genet C Semin Med Genet. (2005) 133C:1–2. 10.1002/ajmg.c.30039 15645523

[6] López P Mosquera F de León J Gutiérrez M Ezcurra J Ramírez F. Suicide attempts in bipolar patients. J Clin Psychiatry. (2001) 62:963–6. 10.4088/JCP.v62n1208 11780877

[7] Dome P Rihmer Z Gonda X. Suicide risk in bipolar disorder: a brief review. Medicina (Kaunas). (2019)55:403. 10.3390/medicina55080403 31344941 PMC6723289

[8] Młodozeniec A Jarema M. Ryzyko samobójstwa szacowane na podstawie skal oceny stanu psychicznego. Przeglad Wazniejszych Narzedzi Badawczych. (2010) 6:54–9.

[9] Brezo J Paris J Turecki G. Personality traits as correlates of suicidal ideation, suicide attempts, and suicide completions: a systematic review. Acta Psychiatr Scand. (2006) 113:180–206. 10.1111/j.1600-0447.2005.00702.x 16466403

[10] Turecki G Ernst C Jollant F Labonté B Mechawar N. The neurodevelopmental origins of suicidal behavior. Trends Neurosci. (2012) 35:14–23. 10.1016/j.tins.2011.11.008 22177979

[11] Hawton K van Heeringen K. Suicide. Lancet. (2009) 373:1372–81. 10.1016/S0140-6736(09)60372-X 19376453

[12] Jollant F Bellivier F Leboyer M Astruc B Torres S Verdier R. Impaired decision making in suicide attempters. Am J Psychiatry. (2005) 162:304–10. 10.1176/appi.ajp.162.2.304 15677595

[13] Hollander E Rosen J. Impulsivity. J Psychopharmacol. (2000) 14:S39–44. 10.1177/02698811000142S106 10888030

[14] Moeller FG Barratt ES Dougherty DM Schmitz JM Swann AC. Psychiatric aspects of impulsivity. Am J Psychiatry. (2001) 158:1783–93. 10.1176/appi.ajp.158.11.1783 11691682

[15] Saddichha S Schuetz C. Impulsivity in remitted depression: a meta-analytical review. Asian J Psychiatr. (2014) 9:13–6. 10.1016/j.ajp.2014.02.003 24813029

[16] Whiteside SP Lynam DR. The Five Factor Model and impulsivity: using a structural model of personality to understand impulsivity. Pers Individual Differ. (2001) 30:669–89. 10.1016/S0191-8869(00)00064-7

[17] Patton JH Stanford MS Barratt ES. Factor structure of the Barratt impulsiveness scale. J Clin Psychol. (1995) 51:768–74. 10.1002/1097-4679(199511)51:6<768::AID-JCLP2270510607>3.0.CO;2-1 8778124

[18] Brown VM Wilson J Hallquist MN Szanto K Dombrovski AY. Ventromedial prefrontal value signals and functional connectivity during decision-making in suicidal behavior and impulsivity. Neuropsychopharmacology. (2020) 45:1034–41. 10.1038/s41386-020-0632-0 32035425 PMC7162923

[19] Pattij T Vanderschuren LJMJ. The neuropharmacology of impulsive behaviour. Trends Pharmacol Sci. (2008) 29:192–9. 10.1016/j.tips.2008.01.002 18304658

[20] Bakhshani N-M. Impulsivity: a predisposition toward risky behaviors. Int J High Risk Behav Addict. (2014) 3:e20428. 10.5812/ijhrba.20428 25032165 PMC4080475

[21] Dalley JW Roiser JP. Dopamine, serotonin and impulsivity. Neuroscience. (2012) 215:42–58. 10.1016/j.neuroscience.2012.03.065 22542672

[22] Dervic K Garcia-Amador M Sudol K Freed P Brent DA Mann JJ. Bipolar I and II versus unipolar depression: clinical differences and impulsivity/aggression traits. Eur Psychiatry. (2015) 30:106–13. 10.1016/j.eurpsy.2014.06.005 25280430

[23] Bora E Yucel M Pantelis C. Cognitive endophenotypes of bipolar disorder: a meta-analysis of neuropsychological deficits in euthymic patients and their first-degree relatives. J Affect Disord. (2009) 113:1–20. 10.1016/j.jad.2008.06.009 18684514

[24] Rote J Dingelstadt A-M-L Aigner A Bauer M Fiebig J König B. Impulsivity predicts illness severity in long-term course of bipolar disorder: a prospective approach. Aust N Z J Psychiatry. (2018) 52:876–86. 10.1177/0004867418783062 29969910

[25] Gonda X Pompili M Serafini G Montebovi F Campi S Dome P. Suicidal behavior in bipolar disorder: Epidemiology, characteristics and major risk factors. J Affect Disord. (2012) 143:16–26. 10.1016/j.jad.2012.04.041 22763038

[26] Perroud N Baud P Ardu S Krejci I Mouthon D Vessaz M. Temperament personality profiles in suicidal behaviour: an investigation of associated demographic, clinical and genetic factors. J Affect Disord. (2013) 146:246–53. 10.1016/j.jad.2012.09.012 23044284

[27] Gilbert AM Garno JL Braga RJ Shaya Y Goldberg TE Malhotra AK. Clinical and cognitive correlates of suicide attempts in bipolar disorder: is suicide predictable? J. Clin. Psychiatry. (2011) 72:1027–33. 10.4088/JCP.10m06410 21813075 PMC4035109

[28] Simon OR Swann AC Powell KE Potter LB Kresnow MJ O'Carroll PW. Characteristics of impulsive suicide attempts and attempters. Suicide Life Threat Behav. (2001) 32:49–59. 10.1521/suli.32.1.5.49.24212 11924695

[29] Mann JJ Arango VA Avenevoli S Brent DA Champagne FA Clayton P. Candidate endophenotypes for genetic studies of suicidal behavior. Biol Psychiatry. (2009) 65:556–63. 10.1016/j.biopsych.2008.11.021 19201395 PMC3271953

[30] Evenden JL. Varieties of impulsivity. Psychopharmacology (Berl). (1999) 146:348–61. 10.1007/PL00005481 10550486

[31] McHugh CM Lee RSC Hermens DF Corderoy A Large M Hickie IB. Impulsivity in the self-harm and suicidal behavior of young people: a systematic review and meta-analysis. J Psychiatr Res. (2019) 116:51–60. 10.1016/j.jpsychires.2019.05.012 31195164

[32] First MB Donovan S Frances A. Nosology of chronic mood disorders. Psychiatr. Clin. North Am. (1996) 19:29–39. 10.1016/S0193-953X(05)70271-9 8677218

[33] Mann JJ. Neurobiology of suicidal behaviour. Nat Rev Neurosci. (2003) 4:819–28. 10.1038/nrn1220 14523381

[34] Asberg M Träskman L Thorén P. 5-HIAA in the cerebrospinal fluid. A biochemical suicide predictor? Arch Gen Psychiatry. (1976) 33:1193–7. 10.1001/archpsyc.1976.01770100055005 971028

[35] Ludwig B Dwivedi Y. The concept of violent suicide, its underlying trait and neurobiology: a critical perspective. Eur Neuropsychopharmacol. (2018) 28:243–51. 10.1016/j.euroneuro.2017.12.001 29254658 PMC5809305

[36] Cloninger CR Svrakic DM Przybeck TR. A psychobiological model of temperament and character. Arch Gen Psychiatry. (1993) 50:975–90. 10.1001/archpsyc.1993.01820240059008 8250684

[37] Garcia D Lester N Cloninger KM Robert Cloninger. C. Temperament character inventory (TCI). In: Zeigler-Hill V Shackelford TK editors. Encyclopedia of Personality and Individual Differences. Cham: Springer International Publishing (2017). p. 1–3.

[38] Mueller ST Piper BJ. The Psychology Experiment Building Language (PEBL) and PEBL test battery. J Neurosci Methods. (2014) 222:250–9. 10.1016/j.jneumeth.2013.10.024 24269254 PMC3897935

[39] Woods DL Wyma JM Yund EW Herron TJ Reed B. Factors influencing the latency of simple reaction time. Front Hum Neurosci. (2015) 9:131. 10.3389/fnhum.2015.00131 25859198 PMC4374455

[40] Bechara A Damasio AR Damasio H Anderson SW. Insensitivity to future consequences following damage to human prefrontal cortex. Cognition. (1994) 50:7–15. 10.1016/0010-0277(94)90018-3 8039375

[41] Faul F Erdfelder E Buchner A Lang A-G. Statistical power analyses using G^*^Power 3.1: tests for correlation and regression analyses. Behav Res Methods. (2009) 41:1149–60. 10.3758/BRM.41.4.1149 19897823

[42] Anguelova M Benkelfat C Turecki G. A systematic review of association studies investigating genes coding for serotonin receptors and the serotonin transporter: II. Suicidal behavior. Mol Psychiatry. (2003) 8:646–53. 10.1038/sj.mp.4001336 12874600

[43] Antypa N Serretti A Rujescu D. Serotonergic genes and suicide: a systematic review. Eur Neuropsychopharmacol. (2013) 23:1125–42. 10.1016/j.euroneuro.2013.03.013 23742855

[44] Reist C Mazzanti C Vu R Fujimoto K Goldman D. Inter-relationships of intermediate phenotypes for serotonin function, impulsivity, and a 5-HT2A candidate allele: His452Tyr. Mol Psychiatry. (2004) 9:871–8. 10.1038/sj.mp.4001495 15037867

[45] Ekinci O Albayrak Y Ekinci AE Caykoylu A. Relationship of trait impulsivity with clinical presentation in euthymic bipolar disorder patients. Psychiatry Res. (2011) 190:259–64. 10.1016/j.psychres.2011.06.010 21724267

[46] Oquendo MA Galfalvy H Russo S Ellis SP Grunebaum MF Burke A. Prospective study of clinical predictors of suicidal acts after a major depressive episode in patients with major depressive disorder or bipolar disorder. Am J Psychiatry. (2004) 161:1433–41. 10.1176/appi.ajp.161.8.1433 15285970

[47] Swann AC Lijffijt M Lane SD Steinberg JL Moeller FG. Increased trait-like impulsivity and course of illness in bipolar disorder. Bipolar Disord. (2009) 11:280–8. 10.1111/j.1399-5618.2009.00678.x 19419385 PMC2723745

[48] Johnson SL Carver CS Tharp JA. Suicidality in bipolar disorder: the role of emotion-triggered impulsivity. Suicide Life Threat Behav. (2017) 47:177–92. 10.1111/sltb.12274 27406282 PMC5788807

[49] Watkins HB Meyer TD. Is there an empirical link between impulsivity and suicidality in bipolar disorders? A review of the current literature and the potential psychological implications of the relationship. Bipolar Disord. (2013) 15:542–58. 10.1111/bdi.12090 23822918

[50] Lima IMM Peckham AD Johnson SL. Cognitive deficits in bipolar disorders: Implications for emotion. Clin Psychol Rev. (2018) 59:126–36. 10.1016/j.cpr.2017.11.006 29195773 PMC6404979

[51] Perrain R Dardennes R Jollant F. Risky decision-making in suicide attempters, and the choice of a violent suicidal means: an updated meta-analysis. J Affect Disord. (2021) 280:241–9. 10.1016/j.jad.2020.11.052 33220560

[52] Hawthorne MJ Pierce BH. Disadvantageous deck selection in the Iowa gambling task: the effect of cognitive load. EJOP. (2015) 11:335–48. 10.5964/ejop.v11i2.931 27247661 PMC4873115

[53] Newman AL Meyer TD. Impulsivity: present during euthymia in bipolar disorder? - a systematic review. Int J Bipolar Disord. (2014) 2:2. 10.1186/2194-7511-2-2 25960939 PMC4424222

[54] Richard-Devantoy S Berlim MT Jollant F. A meta-analysis of neuropsychological markers of vulnerability to suicidal behavior in mood disorders. Psychol Med. (2014) 44:1663–73. 10.1017/S0033291713002304 24016405

[55] Keilp JG Gorlyn M Russell M Oquendo MA Burke AK Harkavy-Friedman J. Neuropsychological function and suicidal behavior: attention control, memory and executive dysfunction in suicide attempt. Psychol Med. (2013) 43:539–51. 10.1017/S0033291712001419 22781400 PMC3767483

[56] Cohen RA. Continuous performance tests. In: Kreutzer JS DeLuca J Caplan B editors. Encyclopedia of Clinical Neuropsychology. New York, NY: Springer (2011). p. 699–701.

[57] Maser JD Akiskal HS Schettler P Scheftner W Mueller T Endicott J. Can temperament identify affectively ill patients who engage in lethal or near-lethal suicidal behavior? A 14-year prospective study. Suicide Life Threat Behav. (2002) 32:10–32. 10.1521/suli.32.1.10.22183 11931008

[58] Joyce PR Light KJ Rowe SL Cloninger CR Kennedy MA. Self-mutilation and suicide attempts: relationships to bipolar disorder, borderline personality disorder, temperament and character. Aust N Z J Psychiatry. (2010) 44:250–7. 10.3109/00048670903487159 20180727

[59] Jylhä PJ Rosenström T Mantere O Suominen K Melartin TK Vuorilehto MS. Temperament, character, and suicide attempts in unipolar and bipolar mood disorders. J Clin Psychiatry. (2016) 77:252–60. 10.4088/JCP.14m09472 26797163

[60] Erić AP Erić I Ćurković M Dodig-Ćurković K Kralik K Kovać V. The temperament and character traits in patients with major depressive disorder and bipolar affective disorder with and without suicide attempt. Psychiatr Danub. (2017) 29:171–8. 28636575

[61] Pawlak J Dmitrzak-Weglarz M Skibińska M Szczepankiewicz A Leszczyńska-Rodziewicz A Rajewska-Rager A. Suicide attempts and clinical risk factors in patients with bipolar and unipolar affective disorders. Gen Hosp Psychiatry. (2013) 35:427–32. 10.1016/j.genhosppsych.2013.03.014 23643033

[62] LoBue C Cullum CM Braud J Walker R Winhusen T Suderajan P. Optimal neurocognitive, personality and behavioral measures for assessing impulsivity in cocaine dependence. Am J Drug Alcohol Abuse. (2014) 40:455–62. 10.3109/00952990.2014.939752 25083938 PMC4448965

[63] Tomassini A Struglia F Spaziani D Pacifico R Stratta P Rossi A. Decision making, impulsivity, and personality traits in alcohol-dependent subjects. Am J Addict. (2012) 21:263–7. 10.1111/j.1521-0391.2012.00225.x 22494229

[64] Cowie ME Kim HS Hodgins DC McGrath DS Scanavino MDT Tavares H. Demographic and psychiatric correlates of compulsive sexual behaviors in gambling disorder. J Behav Addict. (2019) 8:451–62. 10.1556/2006.8.2019.35 31416337 PMC7044634

[65] Izci F Fındıklı EK Zincir S Zincir SB Koc MI. The differences in temperament-character traits, suicide attempts, impulsivity, and functionality levels of patients with bipolar disorder I and II. Neuropsychiatr Dis Treat. (2016) 18:177–84. 10.2147/NDT.S90596 26848266 PMC4723022

[66] Guzman-Parra J Streit F Forstner AJ Strohmaier J González MJ Gil Flores S. Clinical and genetic differences between bipolar disorder type 1 and 2 in multiplex families. Transl Psychiatry. (2021) 11:31. 10.1038/s41398-020-01146-0 33431802 PMC7801527

[67] Sadovnick AD Remick RA Lam R Zis AP Yee IM Huggins MJ. Mood Disorder Service Genetic Database: morbidity risks for mood disorders in 3,942 first-degree relatives of 671 index cases with single depression, recurrent depression, bipolar I, or bipolar II. Am J Med Genet. (1994) 54:132–40. 10.1002/ajmg.1320540208 8074163

[68] Kung C-H Lee S-Y Chang Y-H Wu JY-W Chen S.-L. Chen S.-H.. Poorer sustained attention in bipolar I than bipolar II disorder. Ann Gen Psychiatry. (2010) 9:8. 10.1186/1744-859X-9-8 24576314 PMC2833157

[69] Akiskal HS Pinto O. The evolving bipolar spectrum. Prototypes I. II, III, and IV. Psychiatr Clin North Am. (1999) 22:517–34, vii. 10.1016/S0193-953X(05)70093-9 10550853

[70] Karanti A Kardell M Joas E Runeson B Pålsson E Landén M. Characteristics of bipolar I and II disorder: a study of 8766 individuals. Bipolar Disord. (2020) 22:392–400. 10.1111/bdi.12867 31724302

[71] Martins SS Tavares H da Silva Lobo DS Galetti AM Gentil V. Pathological gambling, gender, risk-taking behaviors. Addict Behav. (2004) 29:1231–5. 10.1016/j.addbeh.2004.03.023 15236828

[72] Ronzitti S Loree AM Potenza MN Decker SE Wilson SM Abel EA. Gender differences in suicide and self-directed violence risk among veterans with post-traumatic stress and substance use disorders. Womens Health Issues. (2019) 29(Suppl. 1):S94–102. 10.1016/j.whi.2019.04.010 31253249

