# Supplementary material for: Impulsivity as a Risk Factor for Suicide in Bipolar Disorder
Source: Front Psychiatry. 2021 Jul 23;12:706933. doi: 10.3389/fpsyt.2021.706933 (PMC8342888; doi:10.3389/fpsyt.2021.706933)
Supplement: Supplementary file 1 [file Table_1.docx]

Supplementary Material

**Tab.1. The most significant correlations with R>0.4.**

| **Correlation** | **R** | **p** |
| --- | --- | --- |
| Impulsivity NS2 & Motor: Motor BIS-11 | 0,5515 | <0.000 |
| Impulsivity NS2 & Motor BIS-11 | 0,4188 | 0.004 |
| Novelty Seeking NS3 & Attentional: Cognitive Instability BIS-11 | 0,4591 | 0.001 |
| Novelty Seeking NS3 & Motor: Motor BIS-11 | 0,5912 | <0.000 |
| Novelty Seeking NS3 & Motor BIS-11 | 0,6087 | <0.000 |
| Novelty Seeking NS3 & Nonplanning - Self-Control BIS-11 | -0,4338 | 0.003 |
| Novelty Seeking NS4 & Motor: Motor BIS-11 | 0,4779 | 0.001 |
| Novelty Seeking NS4 & Motor BIS-11 | 0,4042 | 0.007 |
| Ns TOTAL TCI & Motor: Motor BIS-11 | 0,6402 | <0.000 |
| Ns TOTAL TCI & Motor BIS-11 | 0,5726 | <0.000 |
| Ns TOTAL TCI & Nonplanning - Self-Control BIS-11 | -0,4449 | 0.002 |
| Harm Avoidance HA1 & Attentional: Cognitive Instability BIS-11 | 0,6057 | <0.000 |
| Harm Avoidance HA2 & Nonplanning - Cognitive Complexity BIS-11 | -0,4289 | 0.004 |
| Harm Avoidance HA2 & Nonplanning BIS-11 | -0,477 | 0.001 |
| Harm Avoidance HA2 & Sum BIS-11 | -0,4202 | 0.005 |
| Harm Avoidance HA3 & Attentional: Cognitive Instability BIS-11 | 0,4468 | 0.002 |
| Harm Avoidance HA3 & Nonplanning - Self-Control BIS-11 | -0,4089 | 0.006 |
| Harm Avoidance HA3 & Nonplanning - Cognitive Complexity BIS-11 | -0,4105 | 0.006 |
| Harm Avoidance HA3 & Nonplanning BIS-11 | -0,4492 | 0.002 |
| Harm Avoidance HA4 & Attentional: Cognitive Instability BIS-11 | 0,5811 | <0.000 |
| Ha TOTAL & Attentional: Cognitive Instability BIS-11 | 0,4521 | 0.001 |
| Ha TOTAL & Nonplanning - Cognitive Complexity BIS-11 | -0,4513 | 0.001 |
| Ha TOTAL & Nonplanning BIS-11 | -0,4866 | 0.001 |
| Persistance & Nonplanning - Self-Control BIS-11 | 0,4374 | 0.003 |
| Persistance & Nonplanning BIS-11 | 0,4919 | 0.001 |
| Self-Directedness SD2 & Attentional: Cognitive Instability BIS-11 | -0,4297 | 0.003 |
| Self-Directedness SD2 & Motor: Motor BIS-11 | -0,5372 | <0.000 |
| Self-Directedness SD2 & Motor BIS-11 | -0,424 | 0.004 |
| Self-Directedness SD3 & Attentional: Cognitive Instability BIS-11 | -0,4942 | 0.001 |
| Self-Directedness SD3 & Motor: Motor BIS-11 | -0,4916 | 0.001 |
| Self-Directedness SD3 & Nonplanning - Self-Control BIS-11 | 0,4899 | 0.001 |
| Self-Directedness SD3 & Nonplanning BIS-11 | 0,5127 | <0.000 |
| Self-Directedness SD5 & Attentional: Cognitive Instability BIS-11 | -0,4188 | 0.005 |
| Self-Directedness SD5 & Motor: Motor BIS-11 | -0,5161 | <0.000 |
| Self-Directedness SD5 & Motor BIS-11 | -0,4433 | 0.002 |
| Self-Directedness SD5 & Nonplanning - Self-Control BIS-11 | 0,409 | 0.006 |
| Sd TOTAL & Attentional: Cognitive Instability BIS-11 | -0,5142 | <0.000 |
| Sd TOTAL & Motor: Motor BIS-11 | -0,4708 | 0.001 |
| Sd TOTAL & Motor BIS-11 | -0,4621 | 0.001 |

| **Correlation** | **R** | **p** |
| --- | --- | --- |
| Novelty Seeking NS3 & Corr RT mean PCPT | -0,525067 | <0.000 |
| Novelty Seeking NS3 & Mean RT SRT | -0,427668 | 0.001 |
| Novelty Seeking NS3 & Median RT SRT | -0,417281 | 0.002 |
| Novelty Seeking NS4 & Mean RT SRT | -0,545287 | 0.003 |
| Novelty Seeking NS4 & Median RT SRT | -0,553184 | <0.000 |
| Ns TOTAL TCI & Corr RT mean PCPT | -0,403831 | 0.002 |
| Ns TOTAL TCI & Mean RT SRT | -0,416771 | 0.001 |
| Novelty Seeking NS3 & nB1 IOWA | 0,402154 | 0.004 |
| Reward Dependance RD2 & targ acc rate PCPT | 0,466955 | 0.001 |
| Reward Dependance RD2 & ommission errors PCPT | -0,463732 | 0.001 |
| Self-Directedness SD4 & Median RT SRT | 0,407882 | 0.003 |
| Cooperativeness C1 & delayed responses SRT | 0,463998 | 0.001 |
| Cooperativeness C5 & nA1 IOWA | 0,572777 | <0.000 |
| Self-Transcendent ST1 & change after wing IOWA | -0,453014 | 0.001 |
| Self-Transcendent ST2 & change after win IOWA | -0,465106 | 0.001 |
